# PZT-Actuated and -Sensed Resonant Micromirrors with Large Scan Angles Applying Mechanical Leverage Amplification for Biaxial Scanning

**DOI:** 10.3390/mi8070215

**Published:** 2017-07-06

**Authors:** Shanshan Gu-Stoppel, Thorsten Giese, Hans-Joachim Quenzer, Ulrich Hofmann, Wolfgang Benecke

**Affiliations:** Micro System Technology, Fraunhofer Institute for Silicon Technology, Itzehoe 25524, Germany; thorsten.giese@isit.fraunhofer.de (T.G.); hans-joachim.quenzer@isit.fraunhofer.de (H.-J.Q.); ulrich.hofmann@isit.fraunhofer.de (U.H.); wolfgang.benecke@isit.fraunhofer.de (W.B.)

**Keywords:** micromirror, PZT, piezoelectric, position sensors, biaxial scanning

## Abstract

This article presents design, fabrication and characterization of lead zirconate titanate (PZT)-actuated micromirrors, which enable extremely large scan angle of up to 106° and high frequency of 45 kHz simultaneously. Besides the high driving torque delivered by PZT actuators, mechanical leverage amplification has been applied for the micromirrors in this work to reach large displacements consuming low power. Additionally, fracture strength and failure behavior of poly-Si, which is the basic material of the micromirrors, have been studied to optimize the designs and prevent the device from breaking due to high mechanical stress. Since comparing to using biaxial micromirror, realization of biaxial scanning using two independent single-axial micromirrors shows considerable advantages, a setup combining two single-axial micromirrors for biaxial scanning and the results will also be presented in this work. Moreover, integrated piezoelectric position sensors are implemented within the micromirrors, based on which closed-loop control has been developed and studied.

## 1. Introduction

Either for biomedical [1], automotive [2] or for entertainment uses like pico-projector [3], micromirrors are attract increasing interest due to the miniaturized size, low power consumption and low production cost compared to conventional scanning devices. To drive a microelectromechanical systems (MEMS) mirror there are basically four actuating principles: thermal, magnetic, electrostatic and piezoelectric principles. Thermal micromirrors reach large deflections driven by low driving voltages [4]. Yet the power consumption is high compared to the other three driving principles. Meanwhile, the actuation frequencies are limited by the thermal response time. Electromagnetic actuation delivers high force and requires low driving voltage [5], but the needed external magnets and the electromagnetic interference impair the integration and the availabilities of the micromirrors for many applications. Electrostatic microscanners are realized by established manufacturing technology and provide good mechanical performance [6]. However, for the actuation high driving voltage is required and the comb finger capacitors cause high lateral air damping [7], which demands vacuum packaging to improve the mechanical efficiency [6]. In contrast, piezoelectric materials [8,9,10], for example lead zirconate titanate (PZT), deliver high driving force at low driving voltage, so that piezoelectrically driven micromirrors achieve large deflection even operated under ambient conditions. Therefore, together with the improvement of processing technology of piezoelectric materials, piezoelectrically driven micromirrors are showing obvious advantages. 

For most applications of micromirrors, biaxial scanning is needed to sense surface of objects, environments or displaying images in two dimensions, which can be realized by raster or Lissajous scanning principles [11]. Two-dimensional raster micromirrors have been reported by [3,5,12], where the micromirrors are driven resonantly in one dimension and quasi-statically in the other. Thereby the quasi-statically driven axes have been designed to possess frequencies of 1–2 kHz for lowering the stiffness and reaching large displacement, which can affect the mechanical robustness of the devices severely. The second option for micromirrors reflecting laser spots to fill a rectangle field is Lissajous scanning, where both axes are driven resonantly. Depending on designed frequency difference of the two axes, different Lissajous figures will repeat and fill the field. Previous works show that very good results regarding high light density can be realized, if the two axes have a frequency difference of 60 Hz [13]. However, such low frequency difference causes inevitably so strong mechanical coupling of the two axes, that complex controlling is needed to decouple the biaxial motions [11]. Therefore, despite great integration and sophistication, biaxial micromirrors have yet vulnerabilities. By comparison, the approach of applying two single-axial micromirrors to realize biaxial scanning shows significant advantages of high flexibility and no crosstalk of the two axial motions, which simplifies the controlling [10]. Thus, a setup combining two single axial micromirrors for biaxial scanning and the results will be presented in this work. 

Generally high frequency and large displacement of resonant micromirrors are required for many applications like pico-projector [14]. Also, quasi-statically driven vector micromirrors request high-resonant frequency for better mechanical robustness and low settling time [15]. However, these two requirements are contradictory to each other, since high frequency demands high stiffness, while large displacement requires low stiffness of the devices. So, to achieve these two targets simultaneously is the challenge of constructing micromirrors. One of the focuses of this work is to utilize analytic modelling, where the micromirror plate and actuators are considered as an entire system, to improve the mechanical efficiency for realizing high frequency, large displacement and low consumption. Piezoelectric micromirrors are usually driven by beam actuators, which also influence resonant frequencies and consume power due to their bending motions. Hence, mechanical leverage amplification has been applied for ensuring that the power is primarily consumed for mirror torsion than actuator bending. On the other hand, previous works showed that the maximum achievable scan angles of piezoelectric micromirrors are strongly affected by the breaking strength of the used material, for example poly-Si [16]. Thus, fracture strength of poly-Si has been studied for optimizing the mirror designs. 

At the end of the work, closed-loop control based on PZT position sensors are studied and presented. In [17], closed-loop control for piezoelectric micromirrors based on capacitive sensors was reported. Also integrated piezoresistive sensors were described in [18]. Despite the great sensor sensitivities, hybrid integration of capacitive sensors and expensive fabrication of piezoresistive sensors remain their tradeoffs. In contrast to them, integrated piezoelectric sensors cost no extra fabrication steps and deliver measuring signals with large signal-to-noise-ratio (SNR) as well.

## 2. Modelling and Analysis 

### 2.1. Dynamic Leverage Amplification

**Figure 1:**
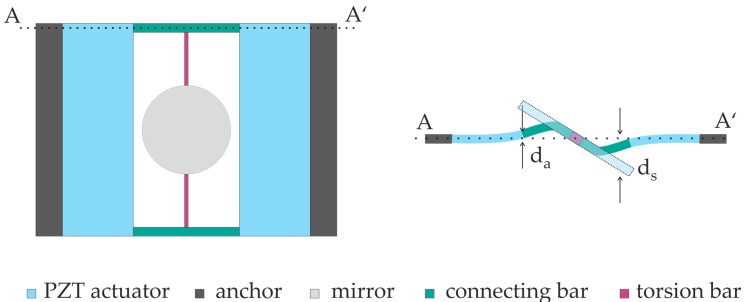
Top view and cross-sectional view of the basic design S. AA’: Cross-section of actuators and connecting bars, *d*_a_ stands for displacement of actuators and *d*_m_ stands for displacement of mirror plate.

The design developments of this work have been strongly supported by finite element method (FEM) simulations. Generally, it is difficult to simulate realistic resonant behaviors of one micromirror except its resonance frequency, since results like achievable displacements are affected by factors like air damping, dielectric and mechanical loss of piezoelectric material, which are complicated to predict accurately. Therefore, static simulations of the designs have been performed for the assessment of the achievable scan angles, since the static behavior equals the border case of strongly damped dynamic behavior. For comparing the achievable displacements of different designs and assessing the efficiency of these designs, a same driving voltage was applied for the static simulations, while the resonant frequencies were calculated by dynamic simulations to evaluate resonant behaviors comprehensively. Before the individual designs were compared with each other, a basic design concept had been developed. Since the focus of the work lies on single-axis micromirrors, the mirror plate is placed in the center of the device, which is linked by torsion bars and connecting bars to two symmetric, surrounding actuators. Figure 1 shows the top view of such a micromirror and the cross-sectional view, which reveals also the principle of such designs: The surrounding piezoelectric actuators are activated by turns. The torque delivered by the actuator is transferred by the connecting bars and torsion bars to rotate the mirror plate. The geometry of the connecting bars has been designed to amplify the displacement of actuators, so that the mirror plate reaches much larger displacements. 

**Figure 2:**
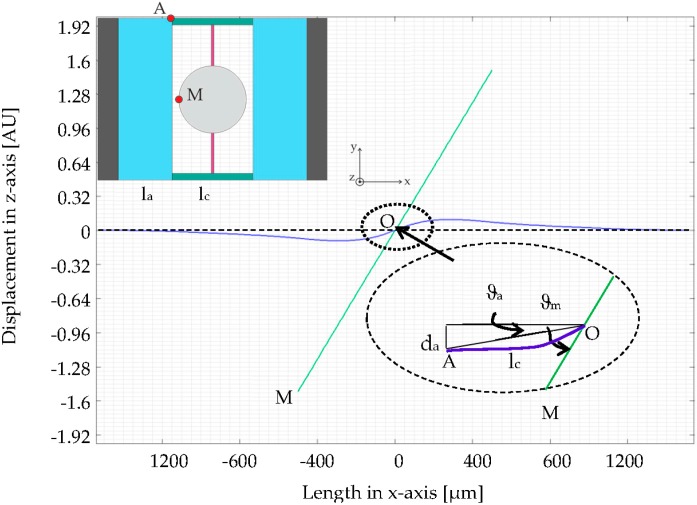
Top view of design S1 with two reference points A and M (A stands at the end of the actuator and M stands on the edge of the micromirror.) and cross-sectional view of a micromirror in the torsional mode, which is calculated by finite element method (FEM) simulations: Deflected actuators and connecting bars (blue) and the torsional mirror plate (green) (O: Center of the mirror plate; *l*_a_: Length of the actuators; *l*_c_: Length of the connecting bars; *ϑ*_a_: Angle of actuators and *ϑ*_m_: Angle of mirror plate).

Actuators, mirror plate, torsion bars and connecting bars can be sorted into two groups: The torsion bars and mirror plate constitute the torsion group (Group T), while the actuators and connecting bars constitute the bending group (Group B). These two groups behave as two coupled oscillators, so that the total energy of the entire system is divided into two parts. Since the actuators possess considerably higher moment of inertia and stiffness than the mirror plate and torsion bars, the mirror plate will show much larger displacement than the actuators in the torsional mode. This is the abovementioned leverage amplification effect and has been proven by FEM simulations using frequency-domain study (Figure 2). 

The angle *ϑ*_a_ is formed by the zero line and OA describing the deflection of the actuator, as Figure 2 shows, while *ϑ*_m_ stands for the torsion angle of the mirror plate. Thereby, the displacement amplification is obviously observed and the ratio *n*_a_ of *ϑ*_m_ to *ϑ*_a_ indicates the amplification efficiency. To calculate this key indicator, *n*_a_ model has been built by using Euler–Lagrange equation [19]:

*L* = *T* − *V* (1)

In Equation (1) *L* stands for the Lagrange function, *T* stands for the kinetic energy and *V* stands for the potential energy. Furthermore, there is a following equation system, which is derived from Equation (1) and applies for the Group T and Group B of the mirror device.

(2) $$\frac{d}{dt}(\frac{\partial L}{\partial {\dot{\vartheta }}_{a}})-\frac{\partial L}{\partial \vartheta_{a}}=M_{a}=M_{\mathrm{PZT}}$$

(3) $$\frac{d}{dt}(\frac{\partial L}{\partial {\dot{\vartheta }}_{m}})-\frac{\partial L}{\partial \vartheta_{m}}=M_{m}=0$$

In Equations (2) and (3) *M*_a_ stands for bending moment of actuators equaling the moment delivered by the PZT *M*_PZT_. Since there is no external force influencing the torsion motion of mirror plate, torsion moment *M*_m_ equals 0. Given the states of kinetic energy *T* and potential energy *V*, torsion angle *ϑ* and angular acceleration $\ddot{\vartheta }$ are depending on the effective mass and effective stiffness of the actuator and the moment of inertia of the mirror plate, Equations (2) and (3) can also be described as [11]:

(4) $$\frac{33}{140}(m_{1}+m_{2}K^{2})l_{c}{}^{2}{\ddot{\vartheta }}_{a}+[(k_{1}+k_{2}K^{2})l_{c}{}^{2}+k_{m}]\vartheta_{a}-k_{m}\vartheta_{m}=M_{\mathrm{PZT}}$$

(5) $$I_{\mathrm{effm}}{\ddot{\vartheta }}_{m}+k_{m}\vartheta_{m}-k_{m}\vartheta_{a}=0$$

In the above equations, the symbols depict the following variables:

*ϑ*_a_:Torsional angle of actuators;*ϑ*_m_:Torsional angle of mirror plate;*m*_1_:Mass of actuators;*m*_2_:Mass of connecting bars;*k*_1_:Stiffness of actuators;*k*_2_:Stiffness of connecting bars;*k*_m_:Stiffness of mirror;*l*_c_:Length of connecting bars;*I*_effm _:Effective moment of inertia of mirror; *M*_PZT_:Torque delivered by PZT;*K*:Geometric matching factor.


According to Fourier Transformation, Equations (4) and (5) can be further described as:

(6) $$\frac{33}{140}(m_{1}+m_{2}K^{2})l_{c}{}^{2}{\dot{\vartheta }}_{a}\cdot j\omega +[(k_{1}+k_{2}K^{2})l_{c}{}^{2}+k_{m}]{\dot{\vartheta }}_{a}/(j\omega )-k_{m}{\dot{\vartheta }}_{m}/(j\omega )=M_{\mathrm{PZT}}$$

(7) $$I_{\mathrm{effm}}{\dot{\vartheta }}_{m}\cdot j\omega +k_{m}{\dot{\vartheta }}_{m}/(j\omega )-k_{m}{\dot{\vartheta }}_{a}/(j\omega )=0$$

**Figure 3:**
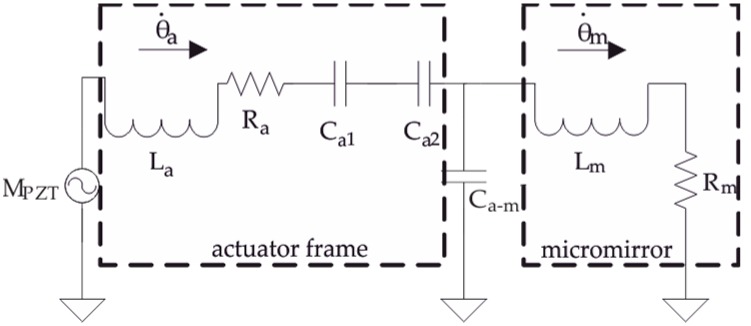
Equivalent electric circuit describing mechanical behavior of micromirrors and actuators.

The Equation system of (6) and (7) can be presented as an equivalent circuit (Figure 3), where $\dot{\vartheta }$ can be considered as the current, the effective mass or moment of inertia, like *I*_effm_, is demonstrated by inductivity L and the inverse of stiffness, like 1/*k*_m_, is demonstrated by capacitance *C*. The electric resistance *R* demonstrates the damping. The values of the circuit components are obtained based on geometry and material parameters of the micromirror design (Table 1). It is to emphasis, using this model the relative energy distribution of different parts (torsional micromirror and bending actuators) can be calculated, the absolute energy dissipation caused by air damping and mechanical losses is not considered. Therefore, the values of *R* are set as 0.

**Figure 4:**
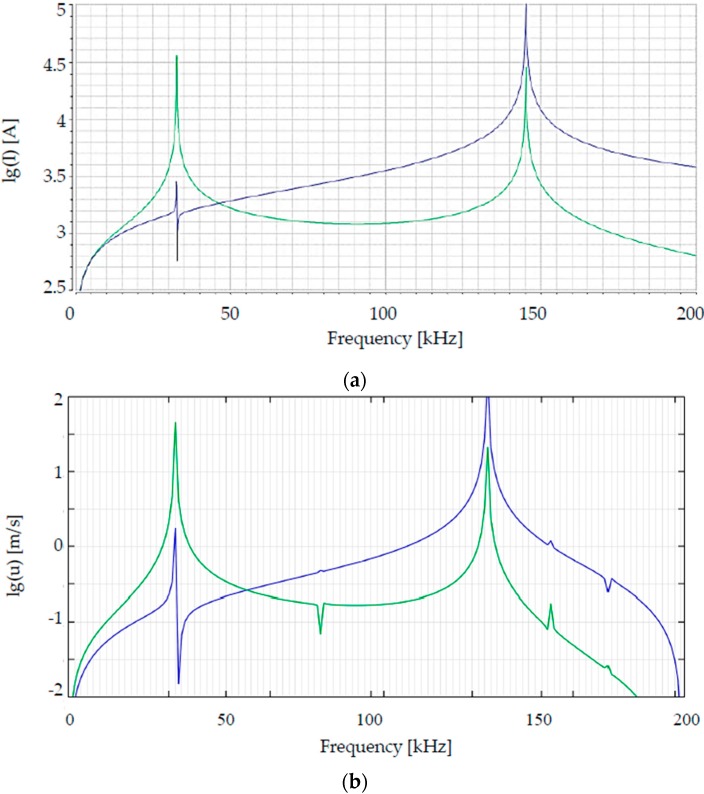
Amplitude spectrums of ${\dot{\vartheta }}_{a}$ (blue) and ${\dot{\vartheta }}_{m}$ (green) of design S1 calculated by (**a**) analytic modelling and (**b**) FEM simulations (I: current of the equivalent circuit and *u*: velocity of actuator and mirror plate.)

The analytic model using the equivalent circuit has been proven by FEM simulations (frequency-domain study), since both results show the identical amplitude spectrums (Figure 4a,b). 

Although no damping mechanisms have been taken into account for the analytic modelling and FEM simulation, bandwidths are apparent at the resonance peaks, as Figure 4a,b show, which means damping. It is caused by different reasons: The damping of analytic modeled results comes from the electric components of the equivalent electric circuit, which has been used for calculation of analytic modelling. FEM simulation results show also damping because of the intrinsic material damping due to the material properties.

The advantage of using this analytic model is to give a clear tendency of influence of every single geometric parameter of the designs, while FEM simulations are accurate, comprehensive but time-consuming. Both the analytic modelling and FEM simulations in Figure 4 have verified the dynamic leverage amplification effect of the design concept. At the resonant frequency of the torsional mode the simulated design, where the first amplitude peak in the amplitude spectrum appears, the displacement of the mirror plate is 32 times as large as that of the actuators, which has been later proven by characterization results. 

### 2.2. Von Mises Stress and Fracture Strength

Due to the dynamic leverage amplification, the PZT delivered torque can be very efficiently used for rotating the mirror plate, so that the micromirror reaches very large scan angles already at low driving voltages and low power consumption. Hence the limitation for reaching larger scan angles for the micromirrors is the fracture strength of poly-Si, of which the micromirrors primarily consist. To investigate the fracture behavior of poly-Si in different micromirrors, these mirrors have been deflected in FEM simulations to a certain rotating angle, which is the maximum achievable rotating angle of these mirrors proven by the characterization results. The observed failure behavior means the calculated maximum Von Mises Stress now equals the fracture strength of this micromirror. The dependence of the fracture strength of polycrystalline materials on their Young’s modules was reported by [20]. Furthermore, in [20,21,22,23] fracture behavior of different polycrystalline materials has been investigated and the fracture strengths lie between 0.6% and 3% of the Young’s moduli of these materials. According to them failure strength of the micromirrors was expected at a mechanical stress level of 1.5 GPa, which approximates 1% of the Young’s modules of used poly-Si.

**Figure 5:**
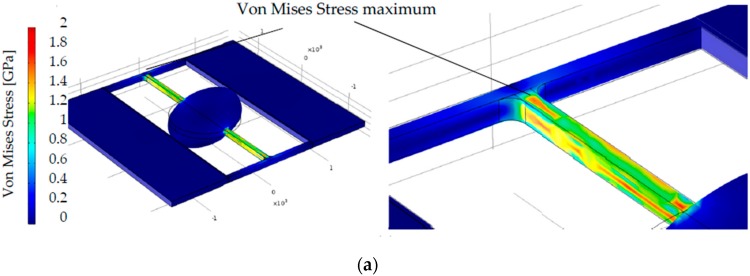

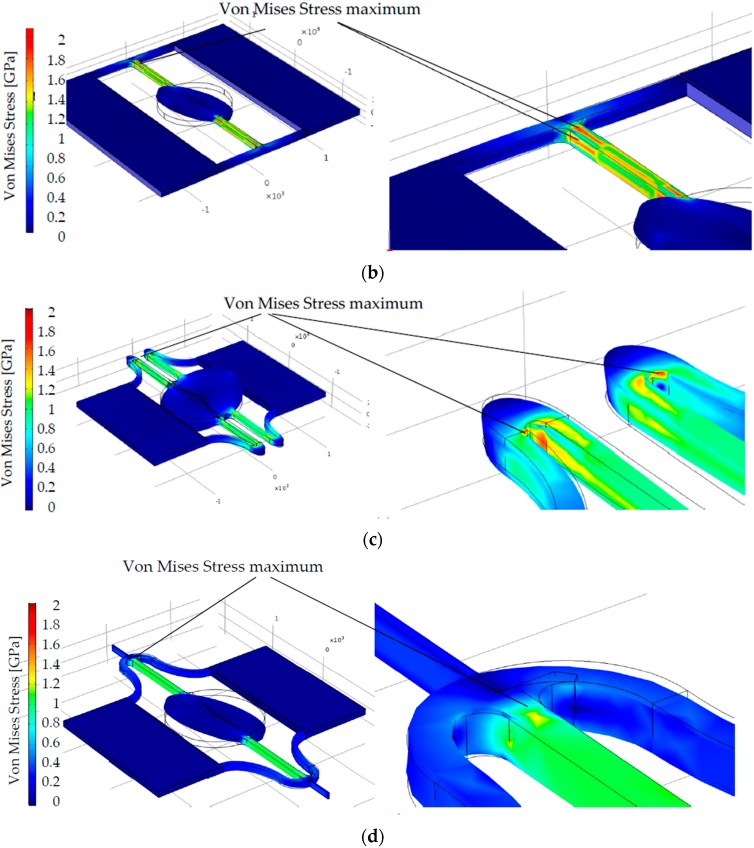
Different maximum Von Mises Stresses in different mirrors, when these mirrors achieve a same mechanical tilting angle of 15°: (**a**) Von Mises stress maximum of 1.8 GPa in design S1; (**b**) Von Mises stress maximum of 1.8 GPa in design S2; (**c**) Von Mises stress maximum of 1.6 GPa in design E5; (**d**) Von Mises stress maximum of 1.4 GPa in design E4.

First of all, the simulation results disclose an important influence of the geometry of micromirrors, especially shapes of the springs, on the maximum Von Mises Stresses. The following pictures in Figure 5 show such a comparison: Different maximum Von Mises Stresses (1.8 GPa, 1.6 GPa and 1.4 GPa) appear within different micromirrors, even if these micromirrors achieve a same mechanical tilting angle of 15°. 

This comparison shows a clear correlation of the maximum Von Mises Stress of a micromirror with its design, for example, meandering and rounding springs can reduce the maximum mechanical stress severely. Additionally, a second finding has been revealed by the later characterization: Although the bearable mechanical stress level was assumed as 1.5 GPa, the micromirrors bear mechanical stress of up to 3.4 GPa and the measurement results show a strong dependence of the fracture strength on the geometry of the designs. Analysis on such correlations and characterization results of these three type micromirrors will be shown in Section 4.2.

## 3. Fabrication

**Figure 6:**
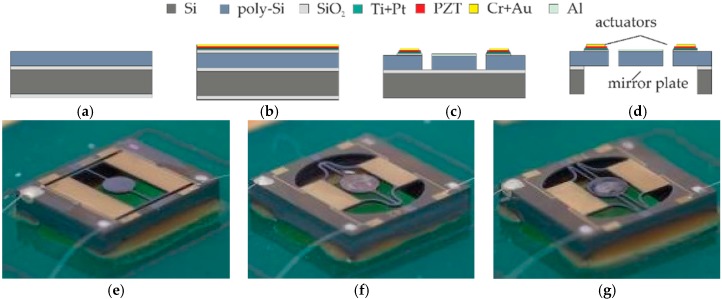
Cross-sectional process flow and device photos: (**a**) Substrate made of poly-Si, SiO_2_ and Si layer; (**b**) Deposition of functional layers; (**c**) Structuring by wet and dry etching from the front side; (**d**) Release by dry etching from the rear side; (**e**) Device photo of design S1; (**f**) Device photo of design E4; (**g**) Device photo of design E5.

For manufacturing of the 1D micromirrors, wafers of 725 µm silicon, 1 µm SiO_2_ and 80 µm epitaxial poly-Si are used as substrates. An additional 1 µm thick SiO_2_ layer on top of the poly-Si is followed by an evaporated thin Ti/Pt layer acting as bottom-electrode and PZT-seedlayer. Subsequently, 2 µm PZT is hot magnetron sputtered featuring a high piezoelectric modulus. On top of the PZT layer, a thin Cr/Au layer serves as top-electrode. After the deposition of all functional layers the Cr/Au layer is wet-etched, while PZT and Ti/Pt layers are dry-etched. Before the 80 µm polysilicon is deep reactive-ion etching (DRIE)-patterned to define the mirrors and actuators, a 100 nm Al layer is deposited as the reflection surface. Finally, the 725 µm silicon and the 1 µm SiO_2_ are etched using DRIE from the rear side to release the mirror. The process flow is illustrated in Figure 6 [10]. 

## 4. Characterization 

### 4.1. Dynamic Behavior

**Figure 7:**
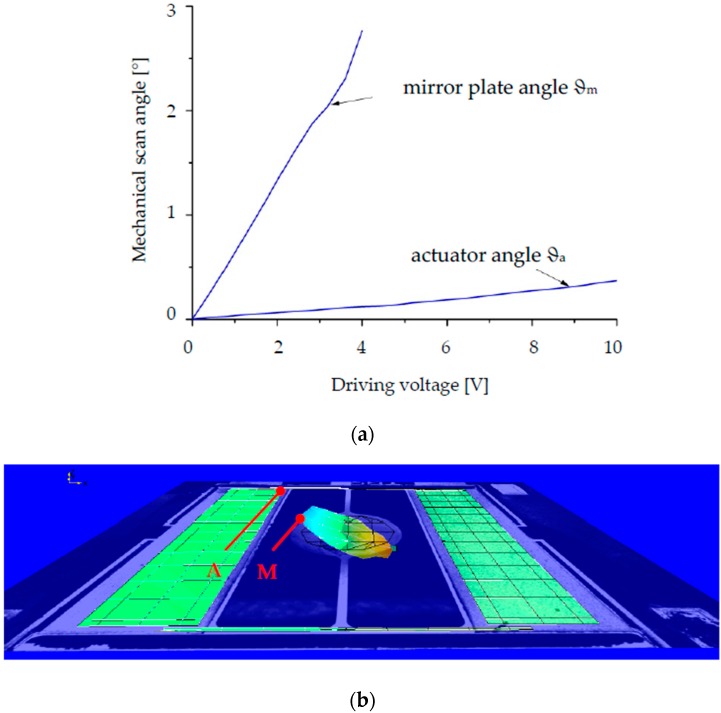
Laser-Doppler-Vibrometry (LDV) measurement results of micromirror with design S1 (measure points are A and M): (**a**) Mechanical scan angles of the mirror plate and the actuators; (**b**) Measurement recording of a deflected micromirror with scan angle of 2.5° measured at point M and the actuators with scan angle of about 0.08° measured at point A.

To verify the dynamic leverage amplification effect of the design concept, as an example the micromirror with design S1 has been measured by a Polytec^®^ Laser-Doppler-Vibrometry (LDV, Polytech Ophthalmologie AG, Zuzwil, Switzerland). As the measurement results in Figure 7a,b show, the mirror plate has much larger displacement than the actuators, when they are driven in the torsional mode. The amplification factor approximates 30, which is identical to the analytic and FEM modelling results shown in Section 2.1. It should be noticed that the LDV measurements have been performed for motion of the mirror plate with a mechanical scan angle of less than about 2.5°, as Figure 7a shows, since the laser spots will be reflected by the rotating mirror plate with larger scan angles out of measurable range of the used LDV lenses. 

### 4.2. Fracture Strength

**Figure 8:**
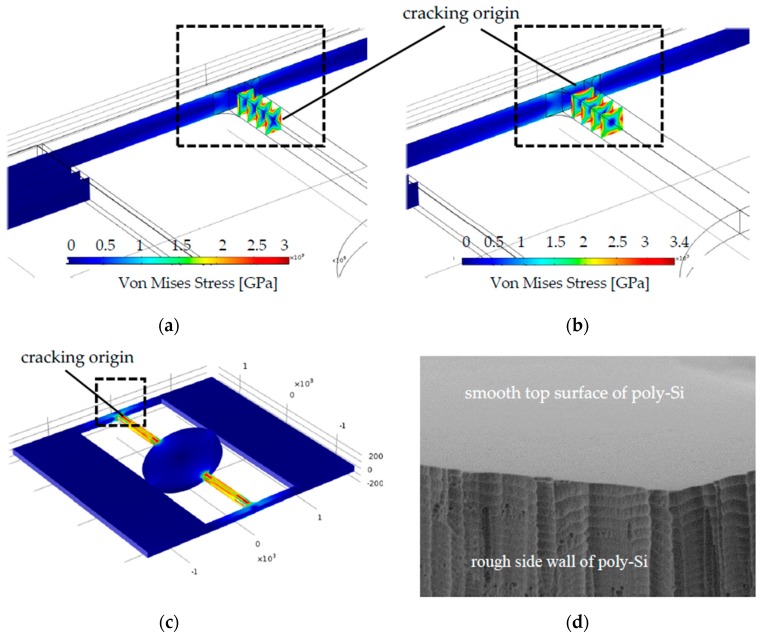
Sectional view of simulation of Design S: (**a**) Design S1 with a rectangular torsion bar (*b*/*t* = 0.75) and the maximum bearable Von Mises Stress is 3 GPa; (**b**) Design S2 with a square torsion bar (*b*/*t* = 1) and the maximum bearable Von Mises Stress is 3.4 GPa; (**c**) Top view of Design S; (**d**) SEM picture of a torsion bar with smooth top surface and rough side wall of poly-Si.

Besides the dependence of the maximum Von Mises Stress of micromirrors on the design, which has been shown by the FEM simulations, the dependence of the fracture strength on the design has been also studied by the characterization results. Two similar designs have been compared in Figure 8a,b, which are based on the basic design S shown in Figure 1. First of all, the cracking origins within the two designs appear both on the torsion bars adjacent to the connecting bars, which can be recognized in Figure 8c. 

Additionally, the only geometry difference between the two designs is the shape of torsion bars. While design S1 has a rectangular torsion bar with a smaller width b than its height t (*b*/*t* = 0.75), design S2 has a square torsion bar with the same width *b* as its height *t* (*b*/*t* = 1) (Figure 8a,b). This only difference results in different distribution of the maximum Von Mises Stress in these micromirrors: The maximum Von Mises Stress of designs S1 appears on the side wall of the torsion bar, whose surface is rough due to the dry etching process, while the maximum Von Mises Stress of designs S2 appears on the polished and smooth top of the torsion bar. Figure 8d shows a SEM image of the torsion bar, where the smooth top surface and the rough side wall can be seen. Due to the notch effect, such rough surface of the side wall benefits the crack formation and crack growth. So, the material bears significantly lower mechanical stress, if the crack origin appears on such tough surfaces [21,22,23]. It is the reason for this phenomenon, that one of the designs can withstand mechanical stress of 3.4 GPa, while the torsion bar of the second micromirror breaks already at 3 GPa, even though both designs are very similar and the main mechanical functional structures (torsion bars, connecting bars, mirror plate and actuators) consist of the same material, poly-Si. 

These findings revealed by the FEM simulations and the characterization give a clear picture of the fracture behavior of the micromirrors. Firstly, the maximum Von Mises Stress is strongly dependent on the designs, and determines at which scan angles the mechanical structures will break. Secondly, also the fracture strength of the micromirrors is dependent on the designs, since the designs have a large influence on the distribution of Von Mises Stress (i.e., where the maximum will occur). 

The characterization results of different four design types in Figure 5 and Figure 8 are shown in the following table (Table 2). 

### 4.3. Setup of Biaxial Scanning Using Two 1D Micromirrors 

**Figure 9:**
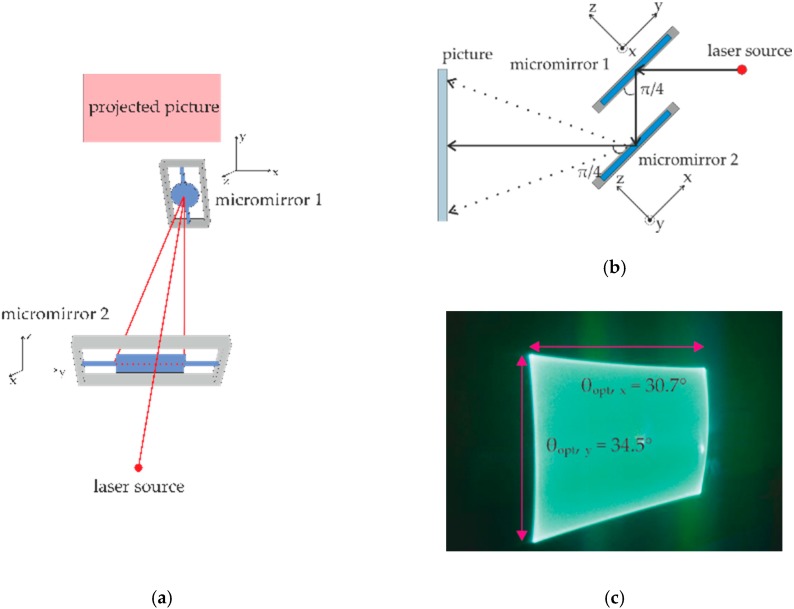
Setup and result of two single-axial micromirrors for 2D scanning: (**a**) Front view; (**b**) Side view; (**c**) Illuminated rectangle.

The above characterization has been conducted to study the mechanical behavior of single-axial micromirrors. Since another focus of this work is to realize biaxial scanning by using two single-axis micromirrors, Figure 9a,b show the construction for this purpose, where two resonant micromirrors have been connected in series [10]. Both micromirrors are parallel to each other with a small distance. The torsion axis of micromirror 1 is inclined to the incident laser beam with an angle of 45°. While micromirror 1 possesses a 1 mm diameter circular aperture, micromirror 2 has a rectangular aperture of 1.4 mm × 4 mm. Both single-axial micromirrors rotate about the own torsion axes, which are perpendicular to each other. The laser beam has been pointed at micromirror 1 and the linear laser beam trajectory of micromirror 1 has been further reflected by micromirror 2 delivering a rectangular light screen. Figure 9c shows such a realized rectangular light screen, which, for example, reaches optical scan angles of 30.7° × 34.5°. 

A rectangle scanned by two independent single-axial micromirrors can possess various length ratios between the horizontal and vertical edges depending on the possible scan angle of the two mirrors. Also, the picture resolution of the displayed pictures, which relates to the frequency ratio [11], can be arbitrarily defined. The flexibility of combing two independent single-axial micromirrors and non-mechanical-crosstalk are the most important advantages of this approach, which simplify the complexity of design, manufacturing and controlling of micromirrors greatly. 

### 4.4. Position Sensing and Closed-Loop Control

**Figure 10:**
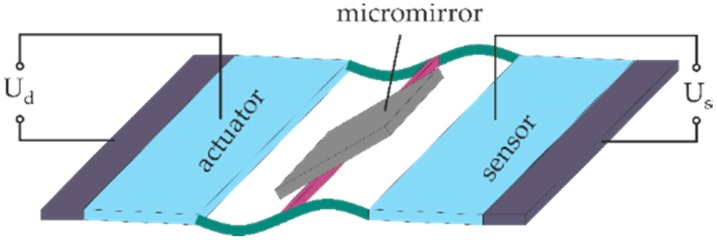
Schematic drawing of realizing micromirror position sensing applying one PZT cantilever as position sensor based on the direct piezoelectric effect (*U*_d_ stands for the driving voltage and *U*_s_ stands for the sensing voltage.)

The last point of this work is to investigate position sensing and closed-loop control of the micromirror. Since the piezoelectric material has the property of converting mechanical energy to electrical energy, one of the position-sensing approaches is to use one of the PZT cantilever as the position sensor, while the second one serves as the actuator, as Figure 10 shows [24]. 

**Figure 11:**
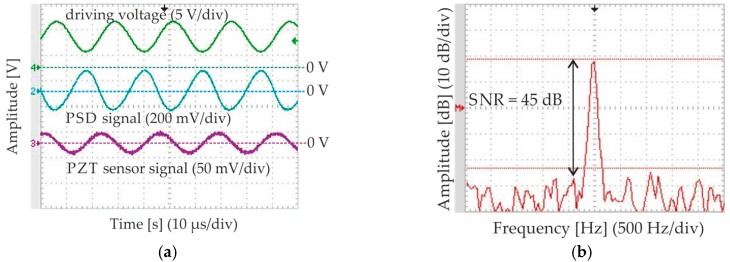
Measurement results of the PZT sensing signal of design S1: (**a**) Comparison of driving voltage (green), PSD position signal (blue) and PZT position signal (violet) in time domain; (**b**) PZT position signal in frequency domain with a SNR of 45 dB after 64 averaging process at a mechanical scan angle of 0.7°. PSD = Position-Sensitive-Device; SNR = signal-to-noise-ratio.

Figure 11a shows the comparison of the driving voltage, position signal of the micromirror measured by a Position-Sensitive-Device (PSD) and a PZT sensing signal in time domain, which are measured on design S1. The result proves a great amplitude correlation between the PZT sensing signal and the PSD signal, which represents the exact mirror position signals. The minor phase shift between the PZT sensing signal and the PSD signal is caused by the capacitance of the PZT actuator and the electrical supply cables of the measurement setup. Additionally, Figure 11b shows the PZT sensing signal in frequency domain. After a simple signal processing of 64 averaging this signal shows already a good signal quality of 45 dB SNR, while the mechanical scan angle was only 0.7°. Since this micromirror can achieve a mechanical scan angle of 26.5°, meaning a full field of view of 106°, the total sensing resolution n is larger than 12 bit, as Equation (8) shows, which can enable the controlling of projecting picture of 1920 pixels.

(8) SNR=1.76+6.02⋅n

**Figure 12:**
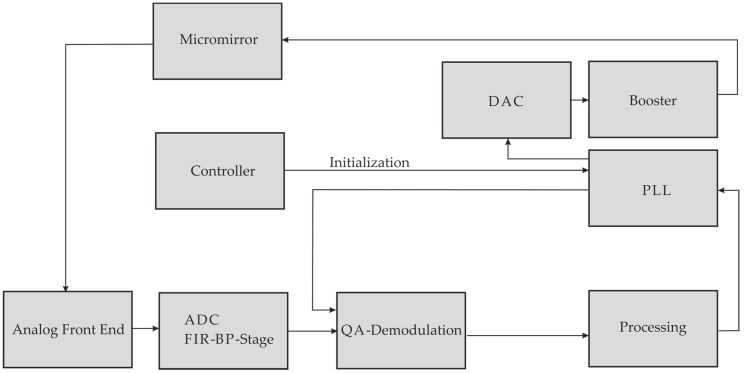
Block diagram of the closed-loop control for the piezoelectrically actuated and sensed micromirror. DAC = Digital-Analog-Converter; ADC = Analog-Digital-Converter; PLL = Phase-Locked-Loop; FIR = Finite-Impulse-Response, BP = Band-Pass.

Based on this great signal quality, closed-loop control has been developed, as Figure 12 demonstrates. The first driving signal is delivered by a controller, which is converted by a Digital-Analog-Converter (DAC) and amplified by a booster, before it reaches the micromirror. Then analog sensing signals from the micromirror are processed by an Analog-Frond-End (AFE) and ADC (Analog-Digital-Converter), before they are demodulated, processed and feed to a Phase-Locked-Loop (PLL), which compose the closed-loop control.

## 5. Conclusions

This work demonstrates the good performance of piezoelectric micromirrors regarding the achievable scan angles and the resonant frequencies. To reach these two targets simultaneously, the mechanical efficiency of the entire system should be raised, which is enabled by the dynamic leverage amplification. This work shows an analytic model to calculate and enlarge the amplification factor by adapting the material and geometrical parameters of micromirrors to increase the mechanical efficiency of the system, whose effect has been confirmed by FEM simulations and characterization results. Furthermore, dependences of the maximum Von Mises Stress and the fracture strength of the micromirrors on the designs have been extensively investigated, to clarify the mechanical fracture behavior and adapt the designs for reaching larger scan angles. 

For demonstrating the advantages of using single-axial micromirrors for biaxial scanning, like high flexibility and no crosstalk, combination setup, integrated piezoelectric position sensors and closed-loop control have been developed and presented. In the future, works for design improvements are projected to achieve larger scan angles, different frequency ratios of the two combined micromirrors for enhancement of the displayed picture quality. Finally, further technology developments for processing piezoelectric materials are also foreseen, to improve the material properties, like the linearity and long-term stability. 

## Figures and Tables

**Table 1:** The electric components and the equivalent mechanical parameters.

Electric Component	*L*_a_	*L*_m_	*C*_a1_	*C*_a2_	*C*_a-m_	*R*_a_, *R*_m_
Equivalent mechanical parameter	$I_{\mathrm{effa}}=\frac{33}{140}(m_{1}+m_{2}K^{2})l_{c}{}^{2}$	$I_{\mathrm{effm}}$	$\frac{1}{(k_{1}+k_{2}K^{2})l_{c}{}^{2}+k_{m}}$	$-\frac{1}{k_{m}}$	$\frac{1}{k_{m}}$	Damping = 0

**Table 2:** The characterization results of Design S1, S2, E4 and E5.

Design	Aperture Diameter [mm]	Driving Voltage Peak-to-Peak [V]	Full Optical Scan Angle [°]	FEM Simulated Frequency [kHz]	Measured Frequency [kHz]
S1	1	22	106.1	35.4	33.3
S2	1	22	106.3	47.1	45.1
E4	1.2	25	86.7	28.1	26.8
E5	1.2	20	104.2	34.1	31.2
